# Viral causes of severe acute respiratory infection in hospitalized children and association with outcomes: A two-year prospective surveillance study in Suriname

**DOI:** 10.1371/journal.pone.0247000

**Published:** 2021-02-19

**Authors:** Amadu E. Juliana, Ming-Jan Tang, Lex Kemps, Albert C. Noort, Sandra Hermelijn, Frans B. Plötz, Rens Zonneveld, Jan C. Wilschut

**Affiliations:** 1 Department of Pediatrics, Academic Pediatric Center Suriname, Academic Hospital Paramaribo, Paramaribo, Suriname; 2 Faculty of Economics and Business, University of Groningen, Groningen, The Netherlands; 3 Department of Medical Microbiology, Academic Hospital Paramaribo, Paramaribo, Suriname; 4 Department of Pediatrics, Tergooi Hospitals, Blaricum, The Netherlands; 5 Department of Pediatrics, Amsterdam UMC, Amsterdam, The Netherlands; 6 Department of Medical Microbiology, University Medical Center Groningen, University of Groningen, Groningen, The Netherlands; NYU Medical Center, UNITED STATES

## Abstract

**Background:**

Viruses are the most frequent cause of severe acute respiratory infection (SARI) in children. It is currently unknown whether presence of a virus, the number of viruses, or type of virus, are associated with clinical outcomes of pediatric SARI in developing countries.

**Methods:**

Between 2012 and 2014 nasopharyngeal swabs and demographic and clinical variables were prospectively collected for surveillance of viral causes of SARI in Surinamese children within 48 hours after hospitalization. These swabs were tested for 18 respiratory viruses using a multiplex polymerase chain reaction (PCR) panel to identify the specific viral causes of SARI, unknown to the treating physicians. In *post hoc* analyses we evaluated if the PCR results, and demographic and clinical characteristics, were associated with course of disease, duration of respiratory support, and length of stay (LOS).

**Results:**

Of a total of 316 analyzed children, 290 (92%) had one or more viruses. Rhinovirus/enterovirus (43%) and respiratory syncytial virus (34%) were most prevalent. Course of disease was mild in 234 (74%), moderate in 68 (22%), and severe in 14 (4%) children. Neither presence of a single virus, multiple viruses, or the type of virus, were different between groups. Prematurity and lower weight-for-age-z-score were independent predictors of a severe course of disease, longer duration of respiratory support, and longer LOS.

**Conclusions:**

Viruses are common causes of pediatric SARI in Suriname, yet not necessarily associated with clinical outcomes. In developing countries, demographic and clinical variables can help to identify children at-risk for worse outcome, while PCR testing may be reserved to identify specific viruses, such as influenza, in specific patient groups or during outbreaks.

## Introduction

Severe acute respiratory infections (SARI) are responsible for an estimated annual 1.4 million deaths among children younger than five years of age worldwide [1]. Approximately 99% of the in-hospital SARI deaths occur in developing countries [1]. Suriname is a developing country in South America with a mortality rate among children younger than five years of 19.6 per 1000 live births. SARI cases are estimated to be responsible for 9% of these deaths [2].

Polymerase chain reaction (PCR) testing has identified many different viral causes of pediatric SARI [3–6]. However, while screening SARI patients with PCR for viral causes has become part of standard care in developed countries, it is often not routinely performed in children in developing countries, since logistics and funding may be challenging. As a result, detailed data from those countries on viral causes of SARI are scarce [1]. In addition, the association of the presence of a specific virus with clinical outcomes is still under debate. For example, presence of multiple viruses is associated with either mild or severe SARI [7, 8]. However, demographic and clinical parameters are known predictors of severe course of disease [9, 10]. To our knowledge, no studies are available from developing countries that assess association of viral causes with outcomes of pediatric SARI.

We performed a prospective surveillance study for identification of viral causes of SARI in Surinamese hospitalized children. Then, we were interested whether presence of a virus, the number of viruses, or specific type of virus, were associated with course of disease, duration of respiratory support, and length of stay (LOS). In addition, we evaluated the potential association of demographic and clinical characteristics of Surinamese children with these outcomes.

## Materials and methods

### Prospective surveillance and inclusion of patients

Between April 2012 and March 2014, surveillance was performed to identify viral causes of pediatric SARI at the Department of Pediatrics of the Academic Hospital Paramaribo (AHP), Suriname. All children (under 18 years of age) admitted to the pediatric ward of the AHP with signs of respiratory infection were eligible to be included into the surveillance database. Inclusion into the surveillance database was based on the WHO case definition for SARI, namely: an acute respiratory illness with onset during the previous ten days requiring overnight hospitalization, along with history of fever or measured fever of ≥ 38°C, and with cough, shortness of breath or difficulty breathing [11]. Before the start of surveillance, we expanded this case definition with the clinical diagnosis of bronchiolitis, since bronchiolitis can present without fever [12]. The attending physician diagnosed bronchiolitis among children less than two years of age in case of a viral upper respiratory tract prodrome followed by signs of acute respiratory distress namely the use of accessory muscles, cough, crackles, and wheezing. Within 48 hours after inclusion a nasopharyngeal swab was taken, which was stored until later analysis, as detailed below. The results from the swab analysis were only used for surveillance purposes and were not known to the treating physicians.

### Sample collection and pathogen detection

Nasopharyngeal swab specimens were collected with Universal Transport Swabs (Copan Italia, Brescia, Italy) from admitted pediatric SARI patients. Both swabs were retained in the same vial and stored at 4°C before daily transport to the laboratory. In the laboratory, swabs were stored in a refrigerator (4°C) for a maximum of 72 hours, before PCR analysis was performed. If longer storage was necessary, the swabs were stored at -20°C. Extraction of genetic material from the samples was performed with a QIAamp MinElute Virus Spin Kit (Qiagen, Hilden, Germany), according to the manufacturer’s protocol. DNA or RNA of influenza A virus (subtype H1N1 and remaining subtypes), influenza B virus, RSV (subtype A and B), parainfluenza virus (subtypes 1–4), adenovirus, rhinovirus/enterovirus (RV/ENT), coronavirus (subtypes OC43, 229E, NL63, HKU1), human metapneumovirus, bocavirus, were detected with the Respifinder SMART 22 multiplex PCR kit (Pathofinder BV, Maastricht, the Netherlands), performed according to the manufacturer’s instructions. For the pre-amplification reaction, a thermocycler (Applied Biosystems, Foster City, USA) was used. The hybridization, ligation/amplification and detection were done with a Lightcycler 480 (Hoffmann-La Roche, Basel, Switzerland). *Mycoplasma pneumonia*, *Chlamydophila pneumonia*, *Legionella pneumophilia*, and *Bordetella pertussis* were also detected with this multiplex PCR kit, but not included into this study on viral causes.

### Data collection

During the surveillance period a standardized case report form was used to collect demographic and clinical data. Patients were admitted through the emergency department or pediatric outpatient clinic. On admission, demographic variables, clinical diagnosis, history of illness, medical history, and findings of physical examination such as respiratory rate, presence of nasal flaring and chest retractions, pulse rate, body temperature (measured axillary or rectal), presence of fever (i.e. defined as temperature ≥38.0°C), transcutaneous hemoglobin oxygen saturation measured with a pulse oximeter with a pediatric sensor, and bodyweight measured with a mechanical scale to the nearest 10 grams, were recorded. Pediatric Early Warning System (PEWS) scores were collected using appropriate vital signs pulse rate and respiratory rate for age [13]. The data were all registered on a standardized case report form. After discharge, the following data were extracted from the medical records in order to complete the case report forms: need for supplemental oxygen therapy, type and duration of respiratory support, the need for intensive-care admission, survival, results of laboratory and microbiological tests taken at admission, diagnosis at discharge and length of stay (LOS). The following laboratory results were collected: hemoglobin, leukocytes and leukocyte subgroups (lymphocytes and neutrophils), and C-reactive protein (CRP). Laboratory tests were not part of the standard care and were only drawn on clinical indication.

### Study cohort and data analyses

Prior to analyses, we excluded cases from the surveillance database that did not meet the WHO SARI criteria, cases in which SARI diagnosis was rejected during admission, and cases in which comorbidity could directly influence the course of disease during the admission, namely in case of congenital heart disease, known immunodeficiency, neuromuscular disorder compromising respiratory function, and congenital respiratory tract malformations. Our first aim was to perform surveillance to identify viral causes of SARI. Our second aim was to evaluate association of the presence of either a single virus or multiple viruses, or the specific type of viruses, with the primary outcome course of disease, and the secondary outcomes duration of respiratory support and LOS in days. Course of disease was based on the maximum respiratory support received and defined as mild (i.e., no respiratory support), moderate (i.e., flow through nasal cannula or non-rebreather mask), or as severe (i.e., nasal continuous positive airway pressure and or invasive ventilation). In addition, we evaluated the association of demographic and clinical variables with these outcomes.

### Statistical analysis

Before statistical analysis, weight was corrected for age using weight-for-age z-scores according to the WHO Growth Standard [14, 15]. Frequencies and percentages of the detected pathogens and clinical parameters were described and calculated. Comparisons of categorical variables between groups were analyzed by chi-square test or Fisher’s exact test. Comparisons of continuous variables between groups were analyzed with one-way independent ANOVA or Student’s t-tests for normally distributed variables and with Mann–Whitney U-test or Kruskal Wallis test for non-normal distributions. Linear regression analysis was used to assess the predictors of days of respiratory support and LOS. Categorical variables were transformed into dummy variables before introduction in the multivariate models. The linear regression correlation was expressed as the standardized coefficient Beta (β) with 95% CI. For all analyses P-values <0.05 were considered significant. All calculations were made using computer software JASP version 0.13.1 (University of Amsterdam, the Netherlands) and Graphpad Prism, Version 8.0.2 (Graphpad Software, Inc.).

### Ethics statement

This study was reviewed and approved by the Surinamese Commission for Human Research (VG014_16). The requirement to obtain informed consent was waived because nasopharyngeal swab sampling was performed for surveillance purposes and considered a noninvasive intervention for children who were hospitalized due to respiratory infection. Collected data were encoded for the researchers.

## Results

### Demographics of the study cohort

**Fig 1:**
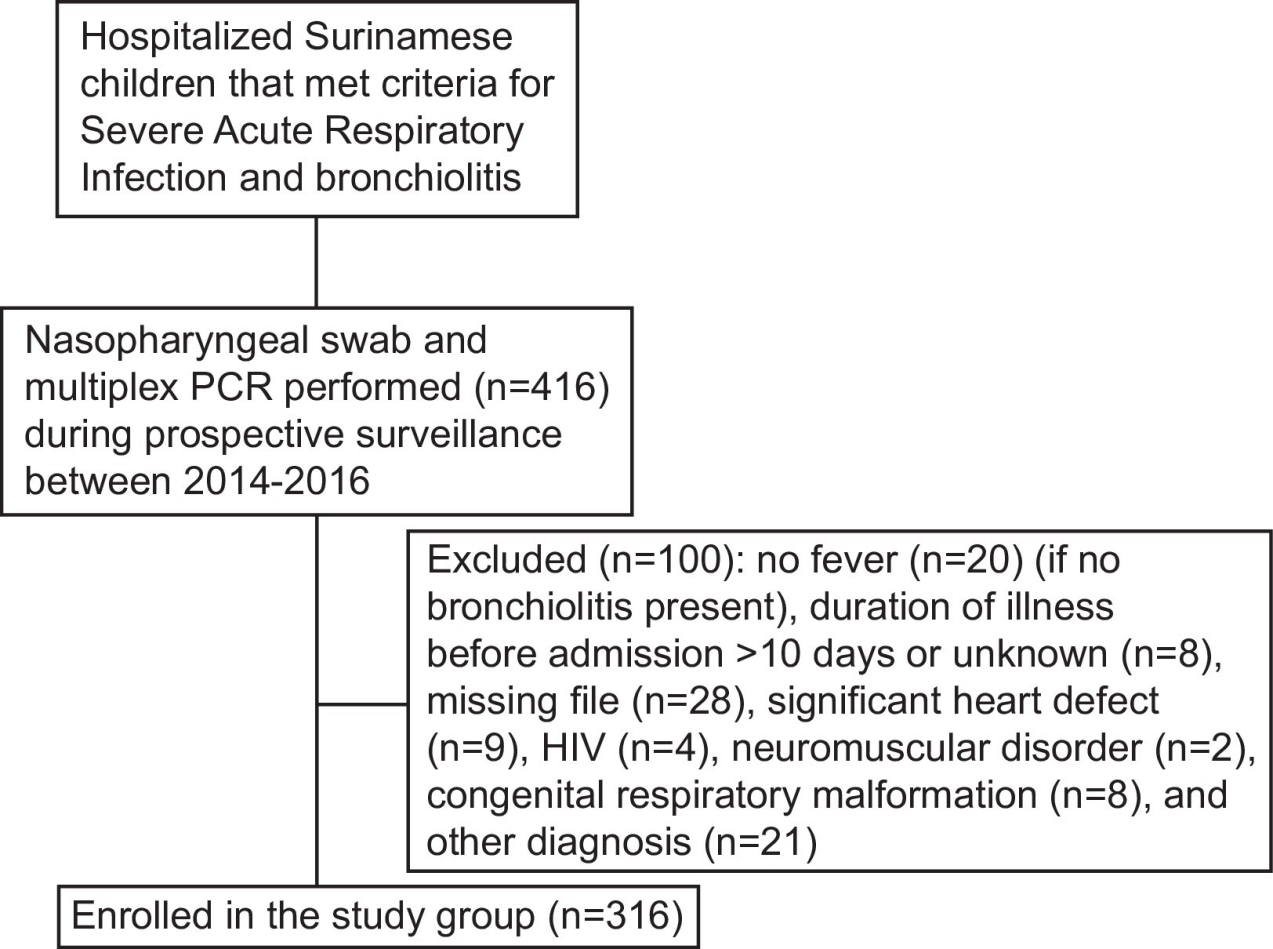
In-and exclusion process.

Nasopharyngeal swab were collected from 416 patients, of whom 100 were excluded (Fig 1). Of 316 included patients 130 (41%) were female. Four children (1.3%) were admitted to the intensive care of whom one (0.3%) died (Table 1).The median age was 8.6 (IQR 3.4–19.5) months. Additional demographic, clinical, and laboratory data are summarized in Table 1.

**Table 1:** Demographics of Surinamese hospitalized children (n = 316) with acute respiratory infections, categorized by maximum respiratory support received.

**Demographic variables**	n (%)		Mild	Moderate	Severe	Total	p-value
			234 (74)	68 (22)	14 (4)	316 (100)	
	Female gender, n (%)		96 (41)	30 (44)	4 (29)	130 (41)	0.559
	Age, months, median (IQR)		9.7 (4.4–21.8)	7.9 (3–15.2)	2.4 (1.5–4.5)	8.6 (3.4–19.5)	**<0.001**
	Prematurity^2^, n (%) (295)		30 (14)	16 (25)	6 (43)	52 (18)	**0.005**
	Weight-age-z-score, mean (SD) (309)		-0.43 (1.25)	-0.85 (1.9)	-0.1.39 (1.6)	-0.53 (1.44)	**0.045**
	Underweight^3^, n (%) (309)		20 (9)	15 (22)	3 (21)	38 (12)	**0.005**
	Ethnicity, n (%) (313)	Maroon	59 (25)	17 (25)	3 (21)	79 (25)	0.871^5^
		Creole	83 (35)	22 (32)	5 (36)	110 (35)	
		Indian	27 (12)	7 (10)	1 (7)	35 (11)	
		Javanese	18 (8)	8 (12)	0	26 (8)	
		Other	47 (20)	14 (21)	5 (36)	66 (21)	
**Clinical variables**	Prior antibiotics, n (%) (264)		36 (19)	12 (20)	1 (9)	49 (19)	0.736
	Fever, n (%) (310)		92 (40)	26 (39)	3 (21)	121 (39)	0.383
	Nasal flaring, n (%) (276)		100 (49)	40 (63)	3 (43)	143 (52)	0.099^6^
	Chest retractions, n (%) (278)		109 (53)	43 (69)	6 (60)	158 (57)	0.071
	*SpO2 admission (%)*, *median (IQR) (216)*		*96 (94–98)*	*91 (88–96)*	*89 (81–95)*	*95 (91–97)*	***<0*.*001***
	*SpO2 <95% on admission n (%) (216)*		*54 (36)*	*38 (66)*	*6 (75)*	*98 (45)*	***<0*.*001***
	Length of stay, days, median (IQR)		3 (2–4)	5 (3–7.3)	9 (6.3–12.8)	2 (1–4)	**<0.001**
	Intensive care admission, n (%)					4 (1.3)	
	Death, n (%)		0	0	1	1 (0.3)	0.263^6^
**Laboratory variables**	Hb (mmol/l), mean (SD) (252)		6.8 (1.0)	6.9 (1.05)	6.6 (0.88)	6.8 (1.0)	0.453
	WBC (*10^9^/l), median (IQR) (252)		13.8 (10–17.9)	14.3 (10.7–17.5)	10.1 (8.4–12.1)	13.6 (10.2–17.6)	0.126
	CRP (mg/dl), median (IQR) (246)		1.6 (0.8–5.3)	1.9 (0.9–6.7)	1.5 (0.3–4.9)	1.8 (0.8–6.4)	0.291
**Virus PCR results**	Positive nasopharyngeal swab, n (%)		211 (90)	65 (96)	13 (93)	289 (91)	0.365
	Single virus detected, n (%)		134 (57)	45 (66)	8 (57)	187 (59)	0.415
	Multiple viruses detected, n (%)		77 (33)	20 (29)	5 (36)	102 (32)	0.830
	Rhinovirus/enterovirus, n (%)		97 (42)	31 (46)	8 (57)	136 (43)	0.459
	Respiratory syncytial virus, n (%)		79 (34)	24 35)	4 (29)	107 (34)	0.888
	Influenza A non-H1N1, n (%)		20 (9)	1 (1)	1 (7)	22 (7)	0.252^5^
	Influenza A H1N1, n (%)		4 (2)	1 (1)	0	5 (2)	1.00^6^
	Influenza B, n (%)		3 (1)	0	0	3 (1)	0.572^6^
	Human metapneumovirus, n (%)		13 (6)	3 (4)	1 (7)	17 (5)	1.00^6^
	Adenovirus, n (%)		25 (11)	5 (7)	2 (14)	32 (10)	0.631
	Parainfluenzavirus, n (%)		29 (12)	10 (15)	2 (14)	41 (13	0.873
	Bocavirus, n (%)		24 (10)	10 (15)	1 (7)	35 (11)	0.525
	Coronavirus n (%)		10 (4)	2 (3)	0	12 (4)	0.738^6^

Mild: no respiratory support. Moderate: flow through nasal cannula or non-rebreather mask. Severe: nasal Continuous Positive Airway Pressure or invasive mechanical ventilation.

^1^ If data was not available for all 316 cases, the numbers between brackets in the second column represent the number for which data were available

^2^ Defined as gestational age below 37 weeks

^3^ Defined as a weight-for-age-z-score of less than -2

^4^ Including indigenous, Chinese, mixed, Caucasian and unknown.

^5^ Moderate and Severe grouped.

^6^ Fisher exact test with Moderate and Severe grouped.

### Viral distribution in the study cohort

A total of 409 viral causes were detected in n = 290 (92%) patient samples that tested positive for at least one virus (Table 2). In 85 (29%) of positive patients, two pathogens were found, in 20 (7%) patients three, and in 1 (0.3%) patient four. The most frequently detected viruses were: RV/ENT (43%), RSV (34%), and parainfluenza virus (13%). RSV was detected in 61 (53%) of the samples collected from young infants between 0–5 months old and was found less frequently in older age categories (P<0.05). Adenovirus was found more frequently in the 12–23 months age group (Table 2). There was no significant difference in the occurrence of co-infections with multiple pathogens across the different age categories (P = 0.221). SARI cases were included all year round. Except for September 2012 and June 2013, RSV was detected during the entire study period. In both consecutive years, we observed that RSV prevalence rates increased from November through May. This pattern was also observed for the other most common viruses RV/ENT, and parainfluenza (S1 Fig).

**Table 2:** Total (n = 409) and age-stratified number of viruses in Surinamese children hospitalized with acute respiratory infection (n = 316).

	Total n (%)^1^	0–5 months, n (%)	6–11 months, n (%)	12–23 months, n (%)	>24 months, n (%)	p-value for comparisons between groups
	n = 316	total n = 114	total n = 74	total n = 64	total n = 64	
Rhinovirus / enterovirus	136 (43)	48 (42)	25 (34)	34 (52)	29 (47)	0.151
Respiratory syncytial virus	107 (34)	61 (53)	24 (32)	14 (22)	8 (13)	**<0.001**^**2**^
Parainfluenza (1,2,3,4)	41 (13)	13 (11)	11 (15)	11 (17)	6 (10)	0.570
Bocavirus	35 (11)	5 (4)	12 (16)	9 (14)	9 (15)	0.151
Adenovirus	32 (10)	3 (3)	10 (14)	14 (22)	5 (8)	**<0.001**^**3**^
Human metapneumovirus	17 (5)	4 (3)	8 (11)	4 (6)	1 (2)	0.075
Coronavirus	12 (4)	6 (5)	1 (1)	3 (5)	2 (3)	0.568
Influenza A virus (non-H1N1)	22 (7)	4 (4)	5 (7)	7 (11)	6 (10)	0.229
Influenza A H1N1	5 (2)	1 (1)	2 (3)	0	2 (3)	
Influenza B virus	3 (1)	1 (1)	2 (3)	0	0	
No virus detected	27 (9)	10 (9)	7 (9)	2 (3)	8 (13)	0.253
Single virus detected	187 (59)	69 (60)	39 (53)	39(60)	40 (65)	0.559
Multiple viruses detected	102(32)	36 (31)	28 (38)	24 (37)	14 (23)	0.221

Due to co-infections, the sum of the proportions of all pathogens will exceed 100%. Proportions are relative to total number of patients per age category.

^1^Proportion relative to total number of patients.

^2^ p <0.001 for comparison group 0–5 months and the rest.

^**3**^p = 0.001 for comparison group 12–23 months and the rest.

### Associations of demographic and clinical characteristics with clinical outcomes

Age, prematurity, weight-for-age-z-score, duration of respiratory support, and LOS were significantly different between groups (Table 1). Neither the presence of a single virus, multiple viruses, or specific types of viruses were different between groups (Table 1). In multiple linear regression analysis with total days of respiratory support as the dependent variable we entered the independent variables of age, gender, weight-for-age-z-score, prematurity and positive viral PCR, and subsequently removed the variables with the highest insignificant P-values except for age. Lower weight-for-age-z-score (β -0.55, 95% CI -0.80 to -0.31, P<0.001) and prematurity (β 0.975 95% CI 0.05 to 1.90, P = 0.039) significantly predicted the amount of days of respiratory support. When LOS (after log transformation to meet assumption of normality of residuals) was the dependent variable, lower weight-for-age-z-score (β -0.134, 95% CI -0.178 to -0.09, P<0.001) and lower age (β -0.004, 95% CI -0.007 to -0.001, P = 0.009) significantly predicted LOS. A positive viral PCR showed a negative coefficient, but did not reach statistical significance (P = 0.07).

## Discussion

In this study, we describe viral causes of SARI and the association of results of PCR testing for these viruses with course of disease and LOS in a large cohort of Surinamese children. Although there was a high prevalence of viral pathogens, we found no association between presence, number, and specific type of the detected viruses and the course of disease, duration of respiratory support, and LOS. However, demographic variables prematurity and lower weight-for-age-z-score were associated with a severe course of disease, and longer duration of respiratory support and LOS.

The overall prevalence of pathogens (92%), and the proportion of viral co-infections (32%) in our study are comparable with those reported in the literature [3, 7, 8, 16, 17]. Consistent with other studies, we found that the predominant pathogens were RV/ENT, RSV, and parainfluenza virus, and that RSV was especially frequently detected among the youngest of children [5, 8, 16, 18–20]. Of the collected samples, 10% tested positive for one of the influenza types. An earlier study observed a 34% prevalence of influenza amongst SARI patients in Suriname [21]. Factors contributing to this difference are the relatively broad definition of SARI used in our study and the fact that our study was limited to the pediatric population, with 80% of patients being less than 2 years of age, and influenza prevalence being lower among children below 2 years of age [22].

Though the burden of RSV disease is mostly in low- and middle-income countries, the understanding of temporal dynamics of RSV disease in these countries has lagged behind [23, 24]. In two consecutive years RSV cases were more prominent in the period from November until March (coinciding with the pattern for the other most common viruses), which only partially overlaps with one of the rainy seasons of Suriname (i.e., from December until January and from April to July). Some, but not all studies in the tropical regions have found RSV seasonal peaks during the rainy season [25–28]. This suggests that in tropical countries, other factors besides rainfall may be relevant.

Bacterial pneumonia, which has a high mortality, was not tested systematically (i.e. with sputum culture or chest X-rays), so that children with bacterial pneumonia could not be excluded from the study. Assuming these children were also in the database, the overall mortality in our study seems low (i.e. 0.3%) considering that 9% of all mortality of children under five years of age in Suriname is explained by SARI according to the WHO [2]. This difference may also be explained by the fact that the WHO definition of mortality in children under-five years of age includes out-of-hospital deaths (e.g., in the rural parts of Suriname), neonatal deaths due to acute respiratory infections, as well as SARI deaths occurring at emergency departments before admission to the pediatric ward.

The clinical utility of PCR testing for viral causes of SARI remains under debate. Our data suggest that the clinical course of SARI may not only be dependent on the presence, number, and type of viruses, but also on host and environmental factors. These data are consistent with those from earlier reports from developed countries [29–33]. Overall, these studies report that multiplex PCR testing for viral causes did not influence admission rates, antibiotic use, antiviral use, and outcomes. In contrast, we found that readily available clinical variables at admission are significant predictors of course of disease, duration of respiratory support, and LOS in our setting. Prematurity and younger age were also found to be significant predictors of severity and LOS in previous studies from our region [10, 34, 35], and low hemoglobin oxygen saturation is also a well-known predictor of severe disease in lower airway infections [36–38]. In developing countries, we suggest to use these clinical variables to identify children at-risk, while PCR testing, which remains financially and logistically challenging, can be reserved for specific patient groups or outbreaks. For example, PCR testing can be used to confirm a specific cause (e.g., influenza virus for which treatment is available or *Bordetella pertussis*, as treatment is available by antibiotics) in specific patients groups (e.g., young infants or hematological patients (e.g., sickle cell) with high risk for mortality due to influenza) and for the identification and monitoring of outbreaks of specific causes (e.g., influenza H1N1). As an example of the latter, shortly after the study period, young infants presented with clear symptoms of *Bordetella pertussis*. Multiplex PCR then confirmed *Bordetella pertussis* in eleven other infants with SARI. In contrast, less than one case of *Bordetella pertussis* had been reported in Suriname each year between 2003 and 2012 [39]. The Department of Public Health was notified and as a response a vaccination program for pregnant women was launched. Last, aside of these immediate clinical purposes, PCR testing can be used to isolate patients with specific causes and for epidemiological reasons. For developing countries, time and cost effectiveness should be evaluated before implementation for such purposes.

The strengths of this study include the prospective collection of data and the fact that PCR results were not available for clinicians, thereby limiting the chance of bias. However, this study is not without limitations. First, a positive PCR result does not differentiate between viable pathogenic viruses or nonviable viral DNA or RNA material after shedding. Second, we did not systematically test for bacterial infection, since this was not ethical due to the invasive nature of the collection of sputum and blood sampling in small children. Last, RSV might exhibit cyclic epidemics over the time span of multiple years. A study period of two consecutive years may not be sufficient to observe these cyclic patterns.

In conclusion, this study contributes to our understanding of the prevalence of viral pathogens associated with children admitted with SARI in Suriname. Our findings underline the importance of viruses as SARI-associated pathogens in this group. We propose that in developing countries, demographic and clinical variables are used to identify children at-risk for severe course of disease. PCR testing can be useful to confirm specific viral causes of SARI in specific patients and during outbreaks.

## Supporting information

S1 Fig Seasonal distribution of Respiratory Syncytial Virus (RSV), rhinovirus/enterovirus (RV/ENT), parainfluenza virus, and total Severe Acute Respiratory Infection (SARI) admissions. (TIF)

## Abbreviations

LOS: Length of Stay
PCR: Polymerase Chain Reaction
PEWS: Pediatric Early Warning Score
RV/ENT: rhinovirus/enterovirus
RSV: Respiratory Syncytial Virus
SARI: Severe Acute Respiratory Infection
WHO: World Health Organization
