# Supplementary figures and images for: Viral causes of severe acute respiratory infection in hospitalized children and association with outcomes: A two-year prospective surveillance study in Suriname
Source: PLoS One. 2021 Feb 19;16(2):e0247000. doi: 10.1371/journal.pone.0247000 (PMC7894877; doi:10.1371/journal.pone.0247000)

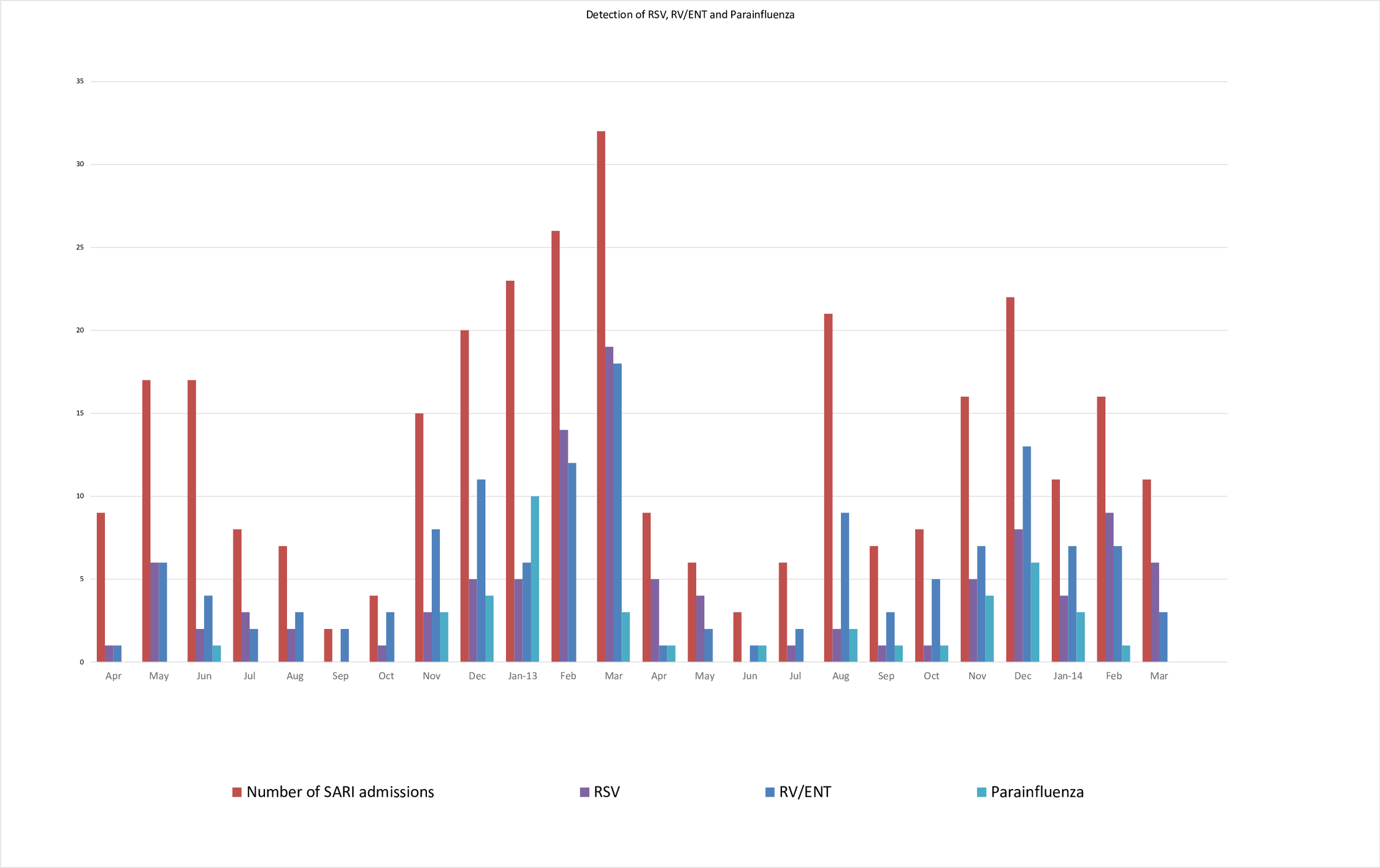

Supplement: S1 Fig — (TIF) [file pone.0247000.s001.tif]
